# H2A.X Phosphorylation in Oxidative Stress and Risk Assessment in Plasma Medicine

**DOI:** 10.1155/2021/2060986

**Published:** 2021-12-13

**Authors:** Clarissa S. Schütz, Matthias B. Stope, Sander Bekeschus

**Affiliations:** ^1^Clinic and Policlinic of Urology, Greifswald University Medical Center, Sauerbruchstr., 17475 Greifswald, Germany; ^2^Clinic and Policlinic of Gynecology and Gynecological Oncology, Bonn University Medical Center, Venusberg-Campus 1, 53127 Bonn, Germany; ^3^ZIK Plasmatis, Leibniz Institute for Plasma Science and Technology (INP), Felix-Hausdorff-Str. 2, 17489 Greifswald, Germany

## Abstract

At serine_139_-phosphorylated gamma histone H2A.X (*γ*H2A.X) has been established over the decades as sensitive evidence of radiation-induced DNA damage, especially DNA double-strand breaks (DSBs) in radiation biology. Therefore, *γ*H2A.X has been considered a suitable marker for biomedical applications and a general indicator of direct DNA damage with other therapeutic agents, such as cold physical plasma. Medical plasma technology generates a partially ionized gas releasing a plethora of reactive oxygen and nitrogen species (ROS) simultaneously that have been used for therapeutic purposes such as wound healing and cancer treatment. The quantification of *γ*H2A.X as a surrogate parameter of direct DNA damage has often been used to assess genotoxicity in plasma-treated cells, whereas no sustainable mutagenic potential of the medical plasma treatment could be identified despite H2A.X phosphorylation. However, phosphorylated H2A.X occurs during apoptosis, which is associated with exposure to cold plasma and ROS. This review summarizes the current understanding of *γ*H2A.X induction and function in oxidative stress in general and plasma medicine in particular. Due to the progress towards understanding the mechanisms of H2A.X phosphorylation in the absence of DSB and ROS, observations of *γ*H2A.X in medical fields should be carefully interpreted.

## 1. Introduction

Since phosphorylated gamma histone H2A.X (*γ*H2A.X) occurs rapidly, abundant, and stoichiometrically with the frequency of DNA double-strand breaks (DSBs), *γ*H2A.X has proven itself as a recognized indicator for radiation-induced DSBs in particular and direct DNA damage in general [1]. Proceeding from th**i**s proportionality in radiobiology, *γ*H2A.X was used as a DNA-associated biomarker and a direct correlate of DSBs in different studies testing chemical and physical treatments, for instance, in the field of oncology [2, 3].

In addition to the oxidative, via reactive oxygen species- (ROS-) induced DNA damage response (DDR), cold physical plasma presents an innovative, promising concept in antitumor therapy [4–6]. A multicomponent system, cold plasma consists of physical emitters, such as ultraviolet (UV) and thermal radiation, and biological-chemical components, including charged particles and various reactive oxygen and nitrogen species (ROS/RNS) [7]. The anticancer effect of medically accredited plasma jet devices [8] has already been proven to a limited extent in small cohort studies in clinical settings of palliation and precancerous [9–11]. Accompanying plasma-induced apoptosis, phosphorylation of H2A.X was observed in an array of in vitro studies [12–31]. These findings led to the hypothesis that direct (oxidative) damage of DNA results from plasma-generated primary ROS [32]. However, in terms of its cellular effect, cold plasma is confirmed as nongenotoxic and nonmutagenic as studies in vitro, in vivo, and long-term follow-up patients suggest [33–36]. This apparent difference calls for a closer look at *γ*H2A.X in cold plasma treatment, where ROS/RNS are generated extracellularly in contrast to known mutagenic agents such as UV and ionizing radiation (IR) generating radicals directly at the DNA.

Cell metabolism, oxidative stress, DNA damage, and apoptosis are often closely related to H2A.X phosphorylation and have not yet been sufficiently investigated independently to characterize the *γ*H2A.X induction as a function of the plasma treatment. For instance, the cytostatic drug doxorubicin induces DSBs and ROS-mediated oxidative stress leading to H2A.X phosphorylation [37]. As shown in numerous studies, damaged DNA and phosphorylated H2A.X correlate. However, this only applies unidirectionally: every DSB results in H2A.X phosphorylation. Yet, the reverse conclusion that every *γ*H2A.X can be traced back to the presence of a DNA DSB is not permissible [38, 39]. Several studies postulate the cause-effect-consequence relationship that primary ROS (cause), generated exogenously after cold plasma treatment, induce DNA damage directly (effect) marked by nuclear *γ*H2A.X (consequence) [13, 15, 20, 25, 32]. Questionable about this causal relationship is whether plasma-mediated ROS after diffusion through the cell membrane, cytosol, and membranes of endoplasmic reticulum (ER) and the nucleus—not to mention the numerous antioxidant enzymes with exceedingly high rate constants towards ROS [40]—are still able to reach DNA to mediate damage directly. Since, apart from the H2A.X phosphorylation, there is no evidence of cold plasma-associated cytogenetic and intranuclear DNA damage, further clarification of the molecular mechanisms on cells and the *γ*H2A.X induction is required to assess the suitability *γ*H2A.X in plasma medical research.

## 2. Histone *γ*H2A.X

### 2.1. Biochemistry

**Figure 1:**
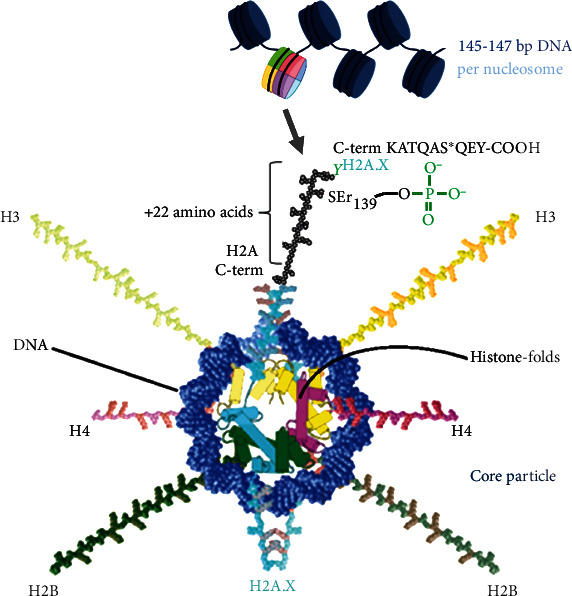
Isoform histone H2A.X in the context of the chromatin structure. The nucleosome is made up of approximately 147 base pairs of DNA around a histone octamer. The core particle can contain instead of H2A.1-H2A.2 two proteins of H2A.X (H2A.X_2_) that are extended c-terminally by the S^∗^QEY-motif of 22 amino acids. The functional group of the posttranslational phosphorylation to *γ*H2A.X is the hydroxyl group (-OH) of the serine at position 139. Model adapted according to [44].

In eukaryotic nuclei, the DNA and DNA-binding proteins and histones form nucleosomes as functional units of the higher-level chromatin complexes (Figure 1) [41]. The crystallographic structure of such a nucleosome comprises 145 to 147 base pairs (bp) of DNA [42], wound as a superhelix around a histone octamer composed of heterodimers from each of the four histone families: H2A, H2B, H3, and H4. Two tetramers, (H2A-H2B)_2_ and (H3-H4)_2_, form the nucleosome core particle [43, 44]. Several genes encode the histones of each family expressed as cell cycle-dependent during DNA replication in the S phase [45]. The histone family H2A as an essential component of chromatin is divided into the members H2A.1-H2A.2, H2A.X, and H2A.Z, of which H2A.1-H2A.2 take the central part [46]. In eukaryotes, H2A.Z represents about 10% of the H2A, H2A.X 2-10% in mammals, and larger fractions up to 25% in lower eukaryotes [2].

H2A.X consists of its primary structure of 142 amino acids and differs from the remaining members of the H2A family by a highly conserved 22 amino acid sequence at the C-terminus, called KATQAS^∗^QEY-COOH, which is not related to any known domain in vertebrate histone H2A [43]. Within the tetrapeptide Ser_139_-Gln_140_-Glu_141_-Tyr_142_ of the S^∗^QEY-motif, histone H2A.X is posttranslationally modified. H2A.X can be phosphorylated C-terminally at position serine_139_ via an ester bond between a phosphate residue and the hydroxyl group (-OH) and is then called *γ*H2A.X [44]. This phosphorylation as an early DDR component is catalyzed by the phosphotransferases ataxia telangiectasia mutated (ATM), the ataxia telangiectasia, and Rad3-related protein (ATR), and the DNA-dependent protein kinase, catalytic subunit (DNA-PKcs) [47, 48]. As serine/threonine kinases, all three of them belong to the phosphoinositide 3-kinases (PI3K). Moreover, mitogen-activated protein kinases (MAPK) p38 and JNK are associated with the formation of *γ*H2A.X [49–51]. PI3K and p38-MAPK are both activated by DNA lesions. Being imperative for a signaling system to perform, it must be possible to switch off the corresponding signals. For this, the H2A.X phosphorylation is reversed by the protein phosphatases PP2A, PP4C, and PP6 and the wild-type p53-induced phosphatase 1 (WIP1) [52, 53].

### 2.2. DDR Signaling

Living organisms are continuously exposed to a wide range of different DNA-damaging agents, affecting health, disease, mutagenesis, and malicious transformation up to cell death. In particular, tumor cells are often deficient in the DDR, so several antitumor therapies are based on the induction of genotoxicity [54]. While DNA single-strand breaks (SSBs) occur 50.000 times per human cell and day, oxidative base damage is less frequent at about 2.000, and intrastrand cross-links and DSBs occur ten times per day [55]. For instance, direct DNA damage is caused by exogenous factors like UV and ionizing radiation, or ROS produced intracellularly by metabolic processes. A variety of repair mediators are integrated into the DNA damage-induced cascade, such as p53 binding protein 1 (53BP1), the tumor suppressor gene product BReast CAncer (BRCA1), ATRIP, KU70/80, and NBS1/Mre11/Rad50 [56–58]. UVB light-emitting radiation between 290 and 315 nm modifies DNA directly by cross-linking between C and T or between two T bases inducing cyclobutene-pyrimidine dimers (CPD) by forming 6-4 photoproducts and inducing DNA single-strand breaks [59]. The SSB repair mechanism is orchestrated by the signaling complex ATR and ATRIP [56–58]. Ionizing radiation is one of the first exogenous agents involved in DSB induction. In response to a radiation-induced DSB, NBS1, Mre11, and Rad50 accumulate and colocalize with ATM and phosphorylated H2A.X [47]. A well-known mechanism upstream of the signaling cascade leading to the phosphorylation of H2A.X is the autophosphorylation of an ATM monomer at serine_1981_ as a result of a DSB [60]. Moreover, the DSB signaling pathway by DNA-PKcs is associated with the protein complex KU70/80 [47]. In one cell cycle, at least 5.000 SSBs are formed caused by endogenous ROS production, and overlapping SSBs lead to DSB formation [39]. Approximately 1% of single-strand breaks are transformed into double-strand breaks, while 99% are repaired impressively, reflecting the importance and efficiency of the cells' DDR system to protect the DNA [61]. As a cellular response to detected DNA damage, a signaling transduction cascade follows in which the cell can be arrested temporarily via checkpoint control activation and permanently by senescence in the cell cycle or in which cell death can be promoted [62].

While PI3K activation initially appears in response to a large number of DNA damage, phosphorylated H2A.X is instead responsible for the association of repair enzymes and signaling molecules, highlighting its diverse and pleiotropic function [57].

### 2.3. Pleiotropic Roles

In addition to its functional role in the nucleosome to ensure genomic stability, (*γ*)H2A.X has a specialized cellular function by signaling and initializing DNA repair [47, 62, 63]. The H2A.X phosphorylation is considered one of the first reactions in the cellular DDR, particularly to DNA DSBs, which are among the DNA lesions with the most pronounced cytotoxicity [64, 65]. *γ*H2A.X has already been used as an indicator of apoptosis by chemotherapeutic agents and an early marker for DSBs in human lung adenocarcinoma cells A549 upon exposure to tobacco smoke [61, 66, 67]. The *γ*H2A.X foci test was reported to identify DNA-damaging agents with the same specificity as the in vitro genotoxicity study standard, the cytokinesis-block micronucleus (MN) assay [68]. In addition to MN formation and mutation frequency, the phosphorylation of H2A.X was defined as one of three genotoxic endpoints. As a de novo modification of the histone H2A.X, *γ*H2A.X has a greater degree of reliability in marking DSBs than other repair proteins present intracellularly even without a DNA lesion, such as 53BP1 [69]. The detection of H2A.X phosphorylation has a great potential in evaluating oncological therapeutic approaches in chemotherapy and radiation therapy. Phosphorylated H2A.X, more precisely the loss of *γ*H2A.X, is suggested as an indicator of radio sensitivity [38, 70, 71].

The formation of *γ*H2A.X is not limited to exposure to ionizing radiation [38]. Several other exogenous noxae lead to H2A.X phosphorylation. Physical factors include UV radiation, low pH, heat, and hypoxia [72–75]. Chemical toxins include DNA-damaging agents such as bleomycin, doxorubicin, and ROS [37]. Even without exogenous noxae, *γ*H2A.X is formed during somatic V(D)J recombination to facilitate antibody variability in B cells or during DNA replication [76, 77]. As part of DNA fragmentation, *γ*H2A.X is integrated into apoptosis, as apoptotic cells are *γ*H2A.X-positive per se [14, 78]. Additionally, phosphorylated H2A.X acts as a tumor suppressor [62]. When employed as a biodosimeter, H2A.X phosphorylation also has relevance in aging research [79]. DSBs accumulate in senescent cells so that *γ*H2A.X acts as an age marker. In turn, and even in the absence of a DSB, phosphorylated H2A.X occurs in mitotic sex chromosomes to form a condensed chromatin domain, the XY body [80]. In cancer research, quantifying *γ*H2A.X evaluates not only the therapy's effectiveness but also has the potential to detect precancerous lesions and to be a prognostic marker of tumor entities [57, 81, 82]. Here, the *γ*H2A.X level reflects cancer-associated genomic instability of the nuclei [83].

In addition to those pleiotropic roles, recent research suggests that the function of *γ*H2A.X is dispensable for the recruitment of DDR enzymes [84]. Neither the initial marking of a DSB nor the initial migration of repair mediators is canceled by the lack of phosphorylated H2A.X so that *γ*H2A.X deficiency does not seem to be deleterious. Phosphorylated H2A.X is said to connect the DDR and ROS signaling pathways [85]. Furthermore, both cascades are involved in apoptosis induction as a consequence of DNA damage. Here, DNA damage-associated *γ*H2A.X regulates Rac1/NOX1-mediated increase in intracellular ROS concentration [86]. A growing body of evidence supports the assumption that *γ*H2A.X occurrence in correlation or noncorrelation with DNA lesions is far more complex. Generally, low quantities of H2A.X phosphorylation are not associated with DNA DSBs [87]. Furthermore, *γ*H2A.X level increase in untreated, normally proliferating cells when entering cell cycle mitosis (M phase) in the absence of a DNA-damaging agent and consequently any induction of a DDR [88]. This apparently suggests a physiological role of *γ*H2A.X apart from DNA damage. In nonstressed cells, downregulation of H2A.X expression leads to damage in the mitochondria [89]. Moreover, the number of *γ*H2A.X foci in apoptotic cells is ten times higher than in nonapoptotic counterparts [90]. Ultimately, there are reports that ROS are responsible for *γ*H2A.X induction so that phosphorylated H2A.X fulfills a potential role in redox signaling [60].

## 3. H2A.X in Radiobiology

### 3.1. *γ*H2A.X Kinetics

The formation of DSBs in tumor cell DNA of tumor tissue is one of the detrimental effects of ionizing radiation, resulting in cell death. Being a biodosimeter in radiobiology, H2A.X is modified within minutes proportionally to the IR intensity, and approximately 1% of all H2A.X proteins is phosphorylated per gray irradiation [91]. One gray induces 35 DSBs per 6 × 10^9^ bp of DNA in the G1 phase of the cell cycle, leading to H2A.X phosphorylation distributed over 1% of the chromatin. According to this, about 0.03% of the chromatin, corresponding to 2 × 10^6^ bp of DNA, are involved per DSB. Immunocytochemically, *γ*H2A.X can be explored using a phosphor-specific antibody targeting the phosphorylation of the C-terminal serine_139_ in the form of so-called *γ*H2A.X foci [39, 92, 93]. It is well observed that the number of foci and radiation-induced DSBs are linked in a one-to-one manner so that the *γ*H2A.X assay is a validated tool for examining the correlation between the absorbed dose and biological effect in radiobiology [38, 47, 94].

It needs to be emphasized that phosphorylated H2A.X should not be equated with *γ*H2A.X foci. As a DDR component, H2A.X molecules are phosphorylated, flanking DNA damage sites. These domains with a local increase in the levels of phosphorylated H2A.X can be detected by immunofluorescence as *γ*H2A.X foci [95]. Using different assays in cell biology, quantification of phosphorylated H2A.X is often based on fluorescence read-out systems [39], and *γ*H2A.X measurements exhibit a wide range of numbers, sizes, and levels of phosphorylation [87]. While *γ*H2A.X foci visualized as bright dots in the nucleus are more specific to DSBs, phosphorylated H2A.X respectively dim *γ*H2A.X foci are also observed in untreated cells. Notably, the H2A.X phosphorylation level includes the DNA damage sites and free form of *γ*H2A.X if measured by global methods such as flow cytometry and western blotting. Hence, different findings might be obtained when comparing total vs. nuclear *γ*H2A.X levels, depending on the method used. Methods other than nuclear foci counting may tend to overestimate *γ*H2A.X levels related to DSBs. Moreover, a constitutive background expression of *γ*H2A.X gradually increases with cell aging towards senescence [96], while especially tumor cells express high endogenous levels, labeled as cryptogenic *γ*H2A.X [97, 98]. In addition, constitutive *γ*H2A.X levels are cell line and treatment agent-dependent [87], and foci formation and spreading at DNA damage sites is not static but dynamic [99, 100]. This is because foci expand over time so that many foci of lesser intensity generate approximately the same signal as a few intense ones. Thus, potential pitfalls to consider when troubleshooting *γ*H2A.X investigations are foci expansion with time, high background staining, or cytoplasmatic staining for phosphorylated H2A.X. It was estimated that the relation of DNA damage-representing *γ*H2A.X foci and nonfoci-labeled *γ*H2A.X is 1 to 10 to up to 1 to 20 in untreated cells [39]. This also applies to UV radiation [101] and should be carefully considered when analyzing *γ*H2A.X. However, the agent- and dose-dependent nuclear-to-cytoplasmic ratio can be reversed, e.g., with IR [102]. Particularly for low doses, nuclear *γ*H2A.X foci represent the overall staining intensity well, while high doses may induce more pronounced cytoplasmic foci staining. Adding to this complexity, a uniform widespread nuclear H2A.X phosphorylation (pan-nuclear *γ*H2A.X) pattern can be observed especially in cells succumbing to replication stress [103].

### 3.2. Reports In Vitro and In Vivo

Because the response of eukaryotic cells to ionizing radiation is highly conserved and mediated by a DNA repair system characterized by early H2A.X phosphorylation, the *γ*H2A.X foci detection is a well-established and sensitive assay to evaluate persistent DNA DSBs [70, 104]. Discussions on genotoxicological studies emphasized that phosphorylated H2A.X has potential in the clinical setting, in particular, to determine the extent of DNA damage in patients undergoing radiotherapy [105]. However, radiation therapy is a modality of anticancer therapy and is vital in diagnostics, such as computer tomography. Hence, the investigation of *γ*H2A.X adjacent to the DSB is of great relevance [106]. In cell lines in vitro, phosphorylation of H2A.X is a measure of radiosensitivity [107, 108]. Fast loss and less retention of *γ*H2A.X are therefore associated with increased radioresistance [38]. Hence, radiosensitive tumor cell lines retain phosphorylated H2A.X longer than radioresistant counterparts, making the former more sensitive to apoptosis than the latter [102]. In addition to various cells in vitro, *γ*H2A.X detection was also used to quantify the effects of IR in tissues exposed ex vivo or in vivo [109, 110]. With a growing range of applications, *γ*H2A.X analysis expands beyond its traditional field of radiobiology, mainly because of its increasingly recognized role in ROS-related conditions.

### 3.3. Role of ROS in Radiotherapy

**Figure 2:**
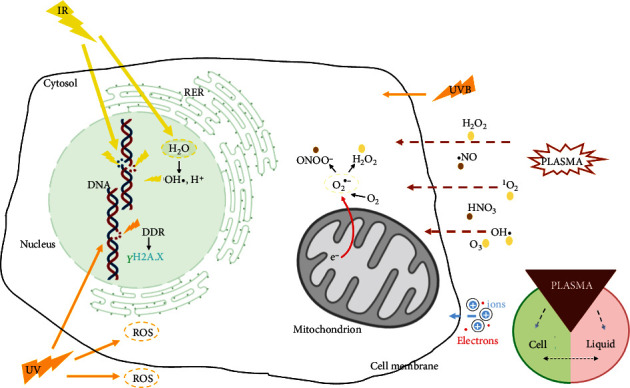
Effects of ionizing radiation versus ultraviolet radiation versus physical plasma. Absorption of ionizing radiation (IR) affects tissue by directly damaging cellular targets and indirectly through water radiolysis, autoamplifying intranuclear ROS production. Similar to IR, UV, especially UVB radiation, can directly damage the DNA. The indirect action of UVB light is majorly mediated by ROS causing oxidative DNA damage, e.g., 8-hydroxy-2′-deoxyguanosine (8-OHdG). Unlike IR and UVB radiation, ROS/RNS need to travel from the gaseous to the liquid phase and eventually to the cells during plasma treatment. ROS accumulate in the extracellular space and at decreased levels in the intracellular environment after selective transit across the plasma membrane to interact with biomolecules in the cytosol.

Radiotherapy damages living systems and tissues ionizing atoms of the irradiated material resulting in changes in the chemical bonds of the irradiated molecules. By cells absorbing IR, organelles like mitochondria or cellular structures, for example, lipids, proteins, and DNA can be directly damaged [47, 111]. Furthermore, IR can cause persistent alteration of atomic components and macromolecules indirectly through radiolysis of water, thereby changing the endogenous redox system by increasing ROS/RNS such as hydroxyl radicals [112]. Moreover, low-dose IR induces mitochondrial ROS production and metabolic oxidative stress (Figure 2) [113, 114]. Both direct and indirect radiation effects initialize molecular signaling pathways that may repair the damage or culminate in base pair deletion, mutation, or cell death [115]. Comparable to IR, UV radiation also has different modes of disruption, especially UVB [116]. In addition to direct DNA modification via CPD-damage, 6-4-photoproducts, and subsequent collision of the replication fork and DNA strand breaks, UVB rays also have an indirect effect on the DNA via photolysis and generation of hydroxyl radicals (OH·) inducing the formation of 8-hydroxy-2′-deoxyguanosine (8-OHdG), a biomarker of oxidative stress [117]. While UV radiation raises ROS levels both extracellularly and intracellularly [59, 118], physical plasma influences the cellular redox equilibrium via exclusively exogenously generated ROS, subsequently exerting oxidative stress intracellularly [119].

## 4. Plasma Medicine

### 4.1. Principle of Cold Physical Plasma

**Figure 3:**
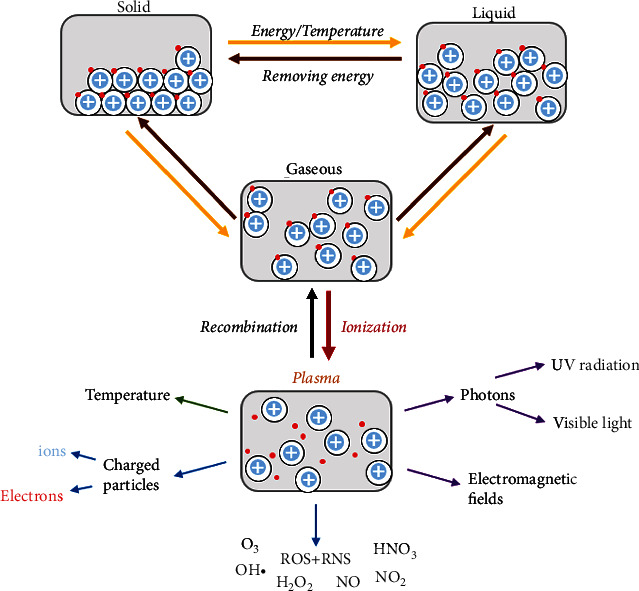
Particle model of the three classical states of matter and active components of the nonclassical fourth state: physical plasma. In the solid state, atoms are held in their position by strong, attractive forces and form a rigid framework. When energy is supplied, the bonds break, the atoms lose their regular order, and the substance liquefies. With a further increase in energy, the atoms lose their cohesion and move freely in space in the gaseous state. Additional energy supply to a gas (ionization) creates gas in an excited state (plasma), a multicomponent system made up of physical and biological-chemical active components. Blue: ions; red: electrons.

Physical plasma is the so-called fourth state of matter after solid, liquid, and gas. By definition, physical plasma is a multicomponent system made of, for instance, electric fields, electrons, ions, thermal and UV radiation, and ROS/RNS (Figure 3). During this phase transition from a gas to the plasma state, electrons are first excited before they dissociate from the atomic shell of the gas molecules. Freely moving electrons and ionized atoms increase the reactivity and electrical conductivity of physical plasmas [7]. With the emission of photons, excited particles return to the ground state, which causes the “plasma glow.” The reactive particle mixture contains charged particles, for example, electrons and ions, excited atoms, and molecules [7, 120]. In addition, due to its ionizing effect and interaction with the surrounding air when operated under atmospheric pressure, physical plasma generates ROS/RNS such as O_3_, H_2_O_2_, ·OH, NO, HNO_3_, O, and ONOO^–^ [121–124]. Thermal movements, especially heavy particles (ions), determine the gas temperature, whereby the plasma temperature correlates with the degree of ionization [7, 125]. In the nonthermal (cold) physical plasma, there is a thermal imbalance in which ions, in contrast to electrons, hardly experience any acceleration (*T*_gas_≙*T*_Ion_ < *T*_e−_ in nonequilibrium plasmas) [7]. With temperatures below 40°C, cold plasma, which is described as partially ionized gas (one particle of 10^9^ being ionized), has the greatest degree of tissue compatibility as it is operated at body temperature. The gas composition, gas humidity, and the surrounding environment are further factors influencing the effect of physical plasmas [122, 124, 126]. Cold plasma is usually generated by applying a high-frequency electrical voltage, and the main generation modes are dielectric barrier discharges (DBD) and jet plasmas [127, 128]. The utilization of cold physical plasma in the field of plasma medicine led to the coining of the term ‘plasma medicine.'

### 4.2. ROS/RNS Generation

While plasma acts physically across the spectrum of its emission, the chemical components, especially ROS/RNS, convey the plasma effects in biological systems. Initially, the cold plasma-derived ROS/RNS are generated exogenously in the gas phase [121]. The species then diffuse or penetrate the liquid cell environment, acting on proteins, lipids, nucleic acids, and other biomolecules. Notably, many cold plasma-generated ROS/RNS are similar to those species occurring in physiological processes, such as metabolism and antimicrobial defense. In vitro, where excess liquid dominates over cell-derived biomass, most short-lived plasma-derived ROS/RNS quickly react to more stable species, such as hydrogen peroxide (H_2_O_2_). Being a nonradical ROS, H_2_O_2_ is an integral mediator of intracellular processes of oxidative eustress and distress, leading to signaling and growth stimulation on the one hand and cellular damage and apoptosis on the other [126, 129, 130]. Recently, the types, sources, gradients, and hormetic cellular effects of cold plasma-derived ROS/RNS in cells and tissues have been summarized [119]. Importantly, it has been noted early on that in vitro plasma-induced toxicity follows known processes in cell death research and redox biology [131]. Accordingly, coping with cold plasma-induced oxidative stress follows the concepts laid out by Helmut Sies, being a primary antioxidative protection system acting through enzymatic and nonenzymatic radical scavengers and antioxidants and a secondary system employing repair mechanisms of the DDR machinery [132]. Even if physical plasma is composed of UV radiation, charged particles, and other components, which may support a synergistic cytotoxic effect, extracellular generated ROS/RNS are the mediators of cold plasma cellular efficacy in the current understanding [133]. For the exogenously derived ROS/RNS to become effective, some species can penetrate cells through aquaporins and via diffusion as well as cholesterol-dependent lipid peroxidation [134]. Intracellular ROS/RNS influence the cascade of the second messenger calcium (Ca^2+^) and vice versa [135, 136]. Both ROS/RNS and calcium are involved in apoptosis signaling, and mitochondria and ER are organelles of crucial relevance. ROS-induced oxidative stress after cold plasma treatment leads to mitochondria oxidation, depolarization of the mitochondrial membrane potential _∆_Ψ_*m*_, and mitochondrial stress [131, 137]. It needs to be emphasized that scavenging exogenous cold plasma-derived ROS/RNS abrogates mitochondrial responses [138], demonstrating the lack of intracellularly generated ROS/RNS with plasma treatment. Moreover, at the ER, plasma-derived ROS cause an overload of calcium in the cytosol and promote ER stress [139]. Via a distinct region called mitochondria-associated ER membranes, the ER is reversibly bound to mitochondria, and the ER stress results in a calcium influx into mitochondria, eventually decreasing the membrane potential [140]. As a result of the _∆_Ψ_*m*_ collapse, the mitochondria disintegration with the subsequent release of cytochrome c triggers apoptosis induction [141].

### 4.3. Therapeutic Successes

The generation of cold plasma has recently developed into an innovative and attractive research field, and its application is the subject of different industries, especially in biophysics, plasma science, and medicine [142]. Physical plasmas are used to modify biorelevant surfaces, for decontamination purposes, and during argon (hot) plasma coagulation to provide hemostasis via cauterization [143, 144]. Moreover, cold physical plasmas at tissue-tolerable temperatures of around 40°C are used therapeutically and directly on the body surfaces due to their antibacterial, anti-inflammatory, and wound healing properties [145]. Other promising fields of application are expected in plastic surgery, oral medicine, and ophthalmology [146–148]. Oncology is an emerging treatment modality with great potency to convey synergy in the multimodal concept of antitumor therapy [6, 149, 150]. The assumption that cold plasma treatment induces apoptosis in cancer cells is supported by observations of various in vitro and in vivo [151–153]. However, a recent large-scale study across 35 cancer lines has shown up to 100-fold differences in the sensitivity of tumor cell lines towards cold plasma [154]. ROS of adequate concentration generally mediates a tumor-suppressive effect, which is used in the medical application of cold plasma in particular. Because cold plasma can interact with the tumor microenvironment, the plasma-mediated modulation of immune cells is of specific interest [155, 156].

**Figure 4:**
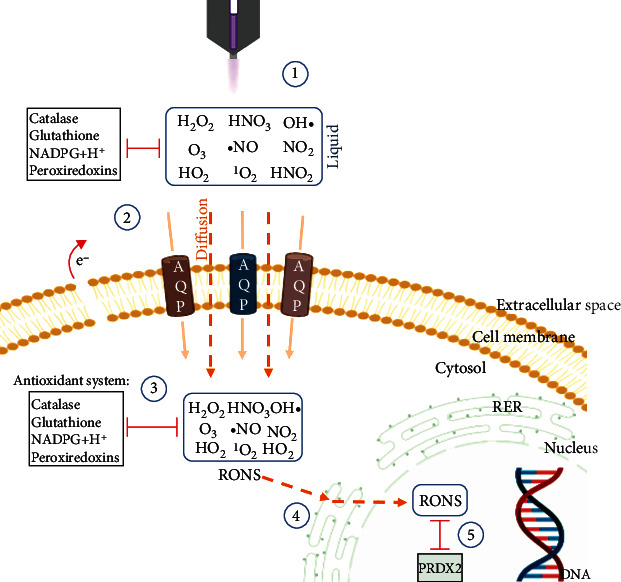
Obstacles of exogenously (cold plasma-generated) short-lived reactive to species from the extracellular to the nuclear compartment. The plasma jet generates ROS/RNS in the liquid phase around the biological system cell ①. Plasma-liquid interactions and components of the antioxidant system are active in the extracellular space. In addition to diffusion, aquaporins enable the limited ROS/RNS passage, especially H_2_O_2_, across the cell membrane ②. Cholesterol-dependent lipid peroxidation by radicals may facilitate transmembrane diffusion through pore formation. Cold plasma exposure may increase the cytosolic ROS/RNS levels exposing the cell to oxidative stress, which the intracellular antioxidant system, including catalase, glutathione, NADPH+H^+^, and peroxiredoxins, attempts to counteract ③. Intracellularly, ROS/RNS would have to pass through several structures and membranes of cell organelles, e.g., the ER, to eventually reach the nucleus ④. Finally, intranuclear antioxidant systems also offer protection from oxidative damage ⑤.

Already before the recent COVID-19 crisis, several studies had examined the efficacy of cold plasma against viral agents along with plasma devices and design concepts for both sanitation and treatment [157]. A plasma source often used in biomedical research of plasma medicine is the plasma jet kINPen, which is operated at atmospheric pressure and consists of a pin electrode in the center of a dielectric capillary and a grounded outer electrode (Figure 4) [158]. Recently, the molecular mechanisms of cold plasma-induced effects were studied more extensively with the kINPen and other cold plasma sources, especially in the light of safety assessment and DNA damage.

## 5. *γ*H2A.X in Plasma Medicine

### 5.1. Summary of Findings

A growing body of literature observed a DDR after cold plasma treatment, including recruitment of ATM, H2A.X respectively *γ*H2A.X, and p53. In the light of the pleiotropic roles of *γ*H2A.X, this led to the misleading conclusion that plasma is mutagenic by causing primary DNA damage in the form of DSBs in living cells [14]. Cold plasma-induced *γ*H2A.X was demonstrated cell type-independent in several cold plasma devices (Table 1) [12, 13, 15–28]. These studies contrast with the results on *γ*H2A.X quantifications in human and porcine skin and oral mucosa ex vivo as well as in vitro genotoxicity and mutagenicity tests according to the OECD (Organization for Economic Co-operation and Development) guideline, none of which had shown any permanent DNA damage after cold plasma exposure [29–31, 33, 34, 159]. So far, only a few investigations in plasma medicine have examined the incidence of cytogenetic damage in cells and tissues using non-*γ*H2A.X assays. However, these observations align with the potentially cytotoxic but neither DNA-damaging nor lasting mutagenic effects of cold plasma [160]. In contrast to UVB radiation, cold plasma-induced *γ*H2A.X did not lead to significantly elevated micronucleus induction, while a correlation between both markers is frequently observed in the field of radiobiology [161, 162]. The lack of a significant increase in the micronucleus frequency as a functional surrogate of genotoxic DSBs is a necessary and sufficient criterion for the hypothesis that cold plasma in plasma medicine has except for—or despite—the H2A.X phosphorylation no sustained genotoxic or mutagenic effect [14, 34]. While this more recent work questioned the unambiguous correlation between *γ*H2A.X and DNA DSBs, the majority of *γ*H2A.X examinations in plasma medicine did not discuss the observation critically that cold plasma-mediated *γ*H2A.X induction does not correlate to DSBs-related MN and do not take into account the interdependence of plasma-generated ROS and phosphorylated H2A.X [87]. Even if the reactive species of intrinsic versus plasma-produced ROS and their cellular targets are fundamentally identical, the theory of plasma-derived ROS directly damaging DNA marked by phosphorylated H2A.X raises doubts at several points along the ROS pathway from extracellular space to the nuclear DNA as explained below (Figure 4). Firstly, cold plasma generates ROS/RNS in the gas phase penetrating the liquid cell environment as an additional barrier to the direct plasma-cell-interaction consisting of an antioxidant system. Moreover, the ROS density produced by the plasma source changes with distance from the active plasma zone. Thus, reaching the cell from the outside, the effects of intracellularly occurring and plasma-derived ROS cannot be assumed to be congruent [122, 126]. Secondly, due to their species-specific reactivity, charge, polarity, and short lifetime, only a fraction of the primary extracellular produced ROS can pass through the plasma membrane, either by passive diffusion or by facilitated processes via transporters or channels, for instance, the H_2_O_2_ transport through aquaporins [163, 164]. Not only the capacity of the transmembrane transport is limited but also the passage depends on the plasma membrane composition, especially on the cholesterol level [134]. Thirdly, the intracellular milieu offers opportunities for interactions between ROS and numerous reactants and ROS scavengers, including catalase, glutathione peroxidase, and peroxiredoxins, which intercept with the ROS on their 2 to 10 *μ*m long cytoplasmatic diffusion pathway from the cell membrane to the nucleus and decrease ROS level [165, 166]. Only stable ROS/RNS such as H_2_O_2_, nitrite, nitrate, and superoxide can passively diffuse across such distances. Notably, H_2_O_2_ is a sturdily nonreactive molecule and reacts with H_2_O_2_-deteriorating enzymes residing at high concentrations in the cytosol 100-1,000 times faster than passive protein oxidation [167]. Fourthly, the remaining ROS must pass through multiple cell organelles and membranes of the ER and ultimately the nucleus. It is unclear how short-lived, reactive species will travel through such protein and lipid-rich environments without finding reaction partners. Fifthly, in the unlikely event such species would make it to the nucleus, there are again antioxidant systems in place in the nucleus to protect against oxidative damage, such as peroxiredoxins 2 (PRDX2) [168]. The argument that H_2_O_2_ could make such travels undisturbed to generate hydroxyl radicals in the Fenton reaction is defective due to the cell's lack of free iron pools. Conversely, iron is bound to storage and proteins, such as ferritin [169]. Moreover, if H_2_O_2_ and not the short-lived cold plasma-derived ROS/RNS would be responsible for DNA damage, it is not cold plasma that is mutagenic but H_2_O_2_. It should be kept in mind that endogenous H_2_O_2_ production during infection control can be exceedingly high, which would lead to the argument that inflammation in wound healing is mutagenic. Altogether, it seems unlikely that short-lived reactive species expelled by cold plasmas are stable and nonreactive enough to travel large distances intracellularly and undisturbed to perform the often-noted DNA-damaging effect.

### 5.2. Role of Signaling and Extracellular ROS

Considering the limitations of short-lived exogenous species directly traveling to the DNA to perform damage, the question remains why *γ*H2A.X induction is documented by many groups, including ours, following cold plasma treatment in vitro. In short, this is due to DNA damage-independent signaling, as the cell naturally activates its protection systems, including *γ*H2A.X, in response to oxidative stress. Because in radiobiology, ionizing radiation-induced DNA damage and oxidative stress always cooccur, there was neither a need nor opportunity to discriminate between in the analysis of H2A.X phosphorylation responses.

The differentiation between H2A.X phosphorylation triggered by primary DNA damage and apoptosis-associated H2A.X phosphorylation as well as the kinetics of *γ*H2A.X formation are decisive for the interpretation of the nuclear *γ*H2A.X occurrence concerning its induction mechanisms. Fluorescence microscopy shows intranuclearly an apoptotic ring that includes H2A.X and DDR proteins in apoptotic cells [170]. Moreover, the *γ*H2A.X presence resulting from a highly damaged DNA is divided into two phases: initially, rapid *γ*H2A.X foci are formed; subsequently, apoptosis is initiated, and the *γ*H2A.X-apoptotic ring is formed. Moreover, agents that do not primarily damage DNA and induce apoptosis, such as ligands of the extrinsic apoptosis initiation pathway or intrinsic stress, induce only the second sequence, the apoptotic ring. Theories about DNA damage by ROS are, on the one hand, a site-specific Fenton reaction and, on the other hand, an intracellular increase in calcium concentration, which in turn activates nucleases [171]. Even though *γ*H2A.X is induced through oxidative stress and DNA can be damaged oxidatively, recent conclusions do not ascribe any primarily caused DNA damage in vitro to the plasma treatment [34, 160]. Moreover, even if lipid peroxidation would have a crucial role in facilitating the transmembrane passage and is a damage mechanism of high-grade oxidative stress, which can contribute to DNA lesions, the oxidative degradation of lipids should not be understood as a critical process of primarily caused DNA damage of exogenous ROS exposure [172–174]. Comparatively, low ROS concentrations and minor quantities of plasma-induced *γ*H2A.X depending on catalase and pan-caspase inhibition refute lipid peroxidation as a potential mechanism for the DSB formation after exposure to plasma-derived ROS [14, 49]. Moreover, if lipid peroxidation were the DNA-damaging mechanism when applying low-dose ROS, the *γ*H2A.X fluorescence should have increased independently of apoptosis and p38-MAPK signaling pathways, which was not the case in our recent study [14].

Although proliferating cells intrinsically have more damaged and unwound DNA owing to DNA and protein synthesis, it cannot be concluded that this makes them more susceptible to ROS-induced nuclear damage [72]. Moreover, albeit different studies formulating the idea that overlapping SSBs lead to DSBs and *γ*H2A.X thus appears at the side of the initial DNA damage after such indirect DSB formation, the conclusion that extracellularly released ROS interact directly with the DNA leading to secondary damage seems less likely than the perspective of a ROS-initiated cytosolic signaling cascade, which results in H2A.X phosphorylation in the nucleus [39, 175]. In addition, the classification of Kalghatgi and colleagues of cold plasma-induced H2A.X phosphorylation being ATR-dependent and ATM-independent is contestable [17]. ATM is a deciphered redox-modulated regulator in the signal cascade upstream of H2A.X phosphorylation. While the kinase is phosphorylated at serine_1981_ in response to a DSB, oxidized ATM acts as an active homodimer via covalent intermolecular disulfide bridged to cysteine_2991_, so that ATM has a dual function: it is active towards both DSB and oxidative stress [37, 60].

The evaluation of the cold plasma effects increasingly focuses on ROS/RNS, which perform their function as cellular signaling molecules or cell-damaging radicals depending on the concentration and localization [85]. Moreover, intracellular changes in the redox system are suggested as a significant event during signal transduction of apoptosis [176]. Equally crucial as directly induced cell death triggered by excessive oxidation of proteins, lipids, or nucleic acids is the potential of ROS to regulate the pathways initiated by other apoptotic stimuli. Particularly, ROS type and source influence the intensity of oxidative stress. It is understood that low ROS levels are agents of redox signaling [177–179]. Furthermore, the redox-sensitives caspases (cysteine-dependent-aspartate-specific proteases) and the stress-sensitives MAPK—p38 and JNK—are decoded regulators upstream of *γ*H2A.X induction [49, 180, 181]. In agreement with this, pharmacological inhibition of oxidative stress signaling pathways by, e.g., SB202190 (p38-MAPK inhibitor) and the apoptosis inhibition via Z-VAD-FMK (pan-caspase inhibitor) significantly neutralized the ROS-triggered but not the UV-induced increase in the *γ*H2A.X levels [14], indicating a strong dependence of the *γ*H2A.X induction on the p38-MAPK signal and the caspase activation. Accordingly, with regard to the occurrence of phosphorylated H2A.X after cold plasma-derived extracellular ROS/RNS, redox-regulated mediators and mitochondria are presented as intracellular interfaces between ROS and apoptosis signaling pathways [182, 183]. As indicated by Hampton and colleagues two decades ago, intracellular ROS and apoptosis are dependent on caspase activation [184]. Plasma-generated ROS/RNS, especially H_2_O_2_, initiate mitochondria-mediated apoptosis: firstly, by changing the membrane potential and secondly, at the level of mRNA expression through simultaneous up-regulation of H2A.X and proapoptotic genes (Bax) and downregulation of antiapoptotic genes (Bcl-2) leading to caspase activation [12]. It follows that cold plasma triggers the mitochondrial intrinsic apoptosis pathway, which is associated with plasma-induced and redox signaling-associated *γ*H2A.X. Furthermore, redox modulation is ascribed to p38-MAPK, whereby its ROS sensitivity is based apparently on both direct and indirect redox regulation [185]. Direct oxidation of cysteine_162_ activates the p38 molecule and influences its interaction with upstream MAP kinases. The indirect regulation, mediated by ROS, occurs via redox activation of upstream kinases such as ASK1 (apoptosis signal-regulating kinase 1) via redox-mediated inactivation of inhibitory MAPK phosphatases [186]. The p38-regulated phosphorylation of H2A.X is also involved in the epigenetic regulation of the expression of proapoptotic Bim during apoptosis induced by the tyrosine kinase inhibitor imatinib chronic myeloid leukemia cells [50]. Accordingly, imatinib stimulates p38, which induces *γ*H2A.X downstream, whose function in connection with apoptosis is closely related to its phosphorylation at serine_139_. This study by Dong and colleagues even reported that *γ*H2A.X expression or the blocking of the p38-MAPK-associated H2A.X phosphorylation by SB202190 sensitizes K562 leukemia cells to apoptosis, which also indicates a central role of *γ*H2A.X in cell death signaling.

Irrespective of DSBs and apoptotic DNA fragmentation, *γ*H2A.X induction was observed in conditions of UVC exposure, which is interpreted as a reaction to JNK-mediated phosphorylation of H2A.X [51, 101]. In this constellation, the UVC light-induced phosphorylation represents an early apoptotic process initiated before the CAD-mediated DNA fragmentation. Analogously to the JNK activation by UVC light, p38-MAPK can be oxidized, stimulating the CAD-mediated nucleosomal DNA fragmentation leading to *γ*H2A.X [180]. Moreover, maximal *γ*H2A.X induction has been shown two hours posttreatment and thus before the apoptosis time window and the beginning of intermediate apoptosis stages, in which *γ*H2A.X increases dramatically in the course of DNA fragmentation [48, 187]. An occurrence of the nuclear *γ*H2A.X without DNA damage and apoptotic fragmentation forces the idea of a phosphorylated histone H2A.X that is strongly intertwined in the signaling of cell death [188]. Recent data with cold plasma even assign *γ*H2A.X a central role in the signaling of the antioxidant defense system [16]. H2A.X knockout cells showed an increase in endogenous ROS levels, could not recruit any element of the antioxidative response via the transcription factor Nrf2 (nuclear factor erythroid 2-related factor 2), which is typically activated in the cytosol as a result of redox-active species, and were associated with mitochondrial damage [89, 189]. Cold plasma treatment has been shown to trigger the translocation of the activated cytosolic Nrf2 into the nucleus in vitro and in vivo [190, 191]. Therefore, these results put phosphorylated H2A.X in the light of a secondary event of redox and apoptosis signaling rather than a primary consequence of direct short-lived ROS/RNS-induced DNA damage upon cold plasma exposure.

### 5.3. Suitability of *γ*H2A.X as a Risk Marker in Plasma Medicine

**Figure 5:**
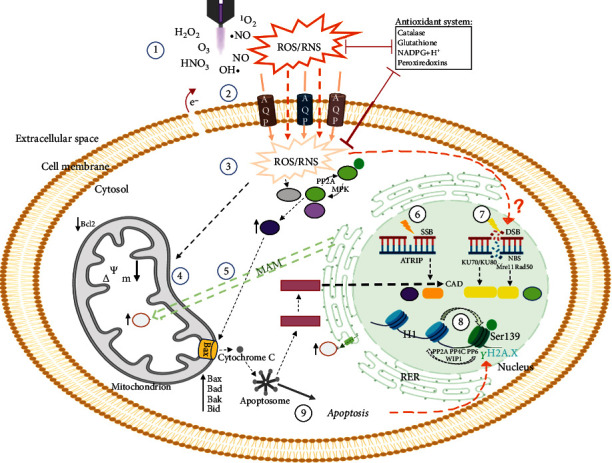
Synopsis of the current understanding of the molecular mechanisms of cold physical plasma effects and the *γ*H2A.X induction in the DDR. A plasma jet generates ROS/RNS in the liquid phase around the cell ①. Aquaporins facilitate the limited ROS/RNS passage across the cell membrane ②. Plasma increases the cytosolic ROS concentration and exposes the cell to oxidative stress, which the intracellular antioxidant system senses ③. By activating mitogen-activated protein kinases (MAPK) and increasing the tumor suppressor protein p53, ROS lead to increased expression of the proapoptotic Bax and cold plasma-induced changes in the mitochondrial membrane potential _∆_Ψ_*m*_ initiating the intrinsic apoptosis pathway ④. Plasma-generated ROS also trigger ER stress, which forces mitochondrial disintegration and increased calcium ⑤. In contrast to plasma, UVB light generates ROS intra- and extracellularly. The direct target of UV and ionizing radiation is DNA. While UVB rays induce single-strand breaks (SSBs) ⑥, IR leads to double-strand breaks (DSBs) ⑦. The DNA damage response elements are PI3 kinases; ATR is associated with SSBs, ATM, and DNA-PKcs are associated with DSBs. All three kinases induce the phosphorylation of the nuclear H2A.X to *γ*H2A.X ⑧. In the current understanding, the plasma-induced DDR, including the activation of PI3 kinases and H2A.X, is not a direct plasma effect on the DNA but rather a consequence of plasma-induced redox signaling and apoptosis ⑨.

The decades-long hypothesis of a causal relationship that *γ*H2A.X represents a reliable indicator for assessing the DNA damage status and that its presence unambiguously marks DSBs is questioned by more and more studies presenting *γ*H2A.X induction in a more diverse light. Similar to the comet assay as a genotoxic endpoint test, H2A.X phosphorylation does not exclusively indicate DNA lesions but also suggests sensitivity to oxidative stress and apoptosis [192]. In all cases, the phosphorylated H2A.X is a biomarker for DSBs but with clear differentiation in the order in which it occurs. In summary, the cause-effect-consequence relationship of *γ*H2A.X formation is best presented in the following: cold plasma-derived exogenous ROS (cause) mediate intracellular oxidative stress that induced apoptosis (effect) whereby the phosphorylated H2A.X marks the secondary DSBs during CAD-mediated DNA fragmentation (consequence). However, this causal chain does not have unlimited coherence. Based on the observation that untreated cells already express the phosphorylated histone at a basal level, the presence of *γ*H2A.X in cells exposed to low-dose oxidative stressors could be interpreted and discussed as a protective entity of antioxidant defense. Without having a long-term toxic effect on the genome, low ROS concentrations (cause) enable the efficiency of repair mechanisms (consequence), which is supported by *γ*H2A.X (effect) [37]. By contrast, increased agent-induced (cause) intracellular *γ*H2A.X levels (effect) might make a cell more sensitive to apoptosis (consequence), and it could have a suppressive effect on the (malignant) transformation of the cell. The *γ*H2A.X assay appears to be less valid for the specific DSB detection and clarification of molecular mechanisms potentially acting via DNA damage, as the phosphorylated H2A.X represents a DDR biomarker of the first generation. As a biosensor of precancerous lesions, tumor processes, and progression, and as a predictor of antitumor therapy efficacy, phosphorylated H2A.X has great potential in prevention, diagnostic, therapy evaluation, and outcome [82, 105]. It can be assumed that oxidative stress promotes tumorigenesis through ROS-mediated proliferation and invasion as well as through endogenous chronic ROS-induced oxidative DNA damage, which could be potentially mapped by increased *γ*H2A.X level as an indicator of genomic instability [193, 194]. However, chronic oxidative stress due to a lack of Nrf2-mediated antioxidant response reduces the H2A.X level through protein degradation, which in turn prevents the normal accumulation of *γ*H2A.X after acute stress [195], making cells more sensitive to cytotoxic and antitumor agents. Yet, this study could not conclude that *γ*H2A.X levels are feasible in risk assessment. Overall, this aligns with another observation showing a clear correlation of *γ*H2A.X and cytotoxicity towards 24 different compounds irrespective of their nature being reportedly mutagenic or nonmutagenic [196]. Consequently, *γ*H2A.X measurements in medical fields and particularly plasma medical research require a careful interpretation of the pleiotropic character of that molecule, including its role in sensing changes in redox homeostasis and apoptotic signaling pathways, are considered and integrated. A mechanistic model is given in Figure 5. Ultimately, *γ*H2A.X does not represent a valid marker for risk assessment in plasma medicine.

## 6. Conclusion

Contrary to treatment with cold plasma, ionizing and UVB radiation-associated *γ*H2A.X is the result of primarily damaged DNA. While the formation of DSB-related micronuclei as genotoxic markers correlates with the nuclear *γ*H2A.X induction for ionizing and UVB radiation but not for cold plasma, plasma but not IR or UVB-light-induced *γ*H2A.X depends on redox-regulated signaling pathways, apoptosis, and caspase activation. IR and UVB act directly on the DNA, after which phosphorylated H2A.X is recruited as part of the DDR, and irreparably damaged DNA promotes apoptosis. However, plasma-derived exogenous and low-dose ROS without sustained genotoxic effects lead to *γ*H2A.X. Consequently, histone H2A.X phosphorylation on serine_139_, initially an indicator of DSBs, does not indicate primarily plasma-induced DNA damage. Physical plasma exerts its biomedical effect through the extracellular release of ROS/RNS, which diffuse intracellularly to exert oxidative stress and induce apoptosis, rather than directly damaging the DNA. Hence, *γ*H2A.X is not the cause but the consequence of cold plasma-induced apoptosis and possibly a protective mechanism to react to oxidative stress and proapoptotic signaling. Due to its pleiotropic roles apart from the DDR and its interlacing action in redox sensing and signaling pathways of apoptosis, phosphorylated H2A.X is less suitable as a risk marker of DSBs in plasma medicine specifically and putatively other medical fields in general.

## Figures and Tables

**Table 1:** Overview of *γ*H2A.X studies in plasma medicine. Both using jet plasmas and dielectric barrier discharge (DBD) as a source of cold physical plasma, *γ*H2A.X was quantified in vitro in low and high malignant cancer cell lines as well as ex vivo in cold plasma-treated tissue.

	Plasma source	Reference
*Low malignant cell line in vitro*		
HEK-293 (human embryonic kidney cells)	Soft jet plasma	Kaushik et al. [12]
MRC-5 (human lung fibroblasts)	Soft jet plasma	Kaushik et al. [12]
Human primary fibroblasts	Plasma needle	Lazović et al. [13]
TK6 cells (B lymphoblastoid cells)	kINPen	Bekeschus et al. [14]
HaCaT cells (human keratinocyte cells)	Jet plasma kINPen	Gaur et al. [15] Schmidt et al. [16]
MCF-10A (human breast epithelial cells)	DBD	Kalghatgi et al. [17]
*Highly malignant line in vitro*		
A549 (lung adenocarcinoma)	Soft jet plasma	Kaushik et al. [12]
T98G (glioblastoma)	Soft jet plasma	Kaushik et al. [12]
U87MG (glioblastoma)	Jet plasma	Gjika et al. [18]
MSK QLL1, SCC1483, SCC15, and SCC25 (squamous cell carcinoma)	Jet plasma	Chang et al. [19]
SCC25 (squamous cell carcinoma)	Jet plasma	Han et al. [20]
Mel (melanoma) SK-MEL 28 (melanoma)	*miniFlat-PlaSter* kINPen	Arndt et al. [21] Sagwal et al. [22]
A549 (lung adenocarcinoma)	DBD	Karki et al. [23]
A2058 (melanoma) B16F10 (melanoma)	DBD	Sensing et al. [24] Kim et al. [25]
HCT116 (colon carcinoma)	DBD	Judée et al. [26] Plewa et al. [27] Choi et al. [28]
*Tissue ex vivo*		
Human skin	(mini)Flat-PlaSter	Isbary et al. [29]
Human oral mucosa	kINPen MED	Hasse et al. [30]
Porcine skin	DBD	Wu et al. [31]
